# USP9X Is Required to Maintain Cell Survival in Response to High-LET Radiation

**DOI:** 10.3389/fonc.2021.671431

**Published:** 2021-07-01

**Authors:** Catherine M. Nickson, Maria Rita Fabbrizi, Rachel J. Carter, Jonathan R. Hughes, Andrzej Kacperek, Mark A. Hill, Jason L. Parsons

**Affiliations:** ^1^ Cancer Research Centre, Department of Molecular and Clinical Cancer Medicine, University of Liverpool, Liverpool, United Kingdom; ^2^ Clatterbridge Cancer Centre NHS Foundation Trust, Bebington, United Kingdom; ^3^ Department of Oncology, CRUK/MRC Oxford Institute for Radiation Oncology, University of Oxford, Gray Laboratories, Oxford, United Kingdom

**Keywords:** centrosome, DNA damage, DNA repair, ionizing radiation, protons, ubiquitin, USP9X

## Abstract

Ionizing radiation (IR) principally acts through induction of DNA damage that promotes cell death, although the biological effects of IR are more broad ranging. In fact, the impact of IR of higher-linear energy transfer (LET) on cell biology is generally not well understood. Critically, therefore, the cellular enzymes and mechanisms responsible for enhancing cell survival following high-LET IR are unclear. To this effect, we have recently performed siRNA screening to identify deubiquitylating enzymes that control cell survival specifically in response to high-LET α-particles and protons, in comparison to low-LET X-rays and protons. From this screening, we have now thoroughly validated that depletion of the ubiquitin-specific protease 9X (USP9X) in HeLa and oropharyngeal squamous cell carcinoma (UMSCC74A) cells using small interfering RNA (siRNA), leads to significantly decreased survival of cells after high-LET radiation. We consequently investigated the mechanism through which this occurs, and demonstrate that an absence of USP9X has no impact on DNA damage repair post-irradiation nor on apoptosis, autophagy, or senescence. We discovered that USP9X is required to stabilize key proteins (CEP55 and CEP131) involved in centrosome and cilia formation and plays an important role in controlling pericentrin-rich foci, particularly in response to high-LET protons. This was also confirmed directly by demonstrating that depletion of CEP55/CEP131 led to both enhanced radiosensitivity of cells to high-LET protons and amplification of pericentrin-rich foci. Our evidence supports the importance of USP9X in maintaining centrosome function and biogenesis and which is crucial particularly in the cellular response to high-LET radiation.

## Introduction

Ionizing radiation (IR) is a major cancer treatment modality for primary and metastatic cancers. Although conventional radiotherapy (e.g. X-rays) is largely employed, there is an increase in the utilization of precision proton beam therapy (PBT) that can more accurately deliver the radiation dose to the tumor, thus limiting any adverse side effects. In particular, PBT is used for the treatment of solid tumors, such as head and neck squamous cell carcinoma (1). The therapeutic effect of IR is predominantly through the induction of DNA damage that ultimately drives cancer cell death. As well as DNA double strand breaks (DSBs), IR causes the formation of complex DNA damage (CDD), which is defined as two or more DNA lesions within one or two DNA helical turns, that contribute to the cell killing effects of the radiation (2–4). This is particularly relevant following high-linear energy transfer (LET) radiation that generates increased quantities and complexity of CDD. Indeed, PBT displays increases in LET at the Bragg peak and at the distal edge where the majority of the radiation dose is delivered, which creates uncertainty in the treatment because of the different biological effects (5). Specifically within cultured cells, CDD has been shown to persist for several hours post-irradiation (6, 7), and therefore, its importance in promoting genome instability and cell death following IR is recognized (5, 8). Although the biological impact of high-LET radiation is largely associated with the direct effects of irreparable CDD induction, this has also been demonstrated to promote disruption and persistent changes to chromatin structure (9, 10), enhance cellular senescence (11), and to increase apoptotic signaling (12). This reflects, to some degree, the potentially diverse mechanisms through which high-LET radiation may act.

The ubiquitin proteasome pathway, catalyzed by E3 ubiquitin ligases and deubiquitylation enzymes (DUBs), is known to be important in the regulation of several DNA repair pathways where it acts to control the cellular levels of key proteins that co-ordinate the repair of the DNA damage (13–15). Recently, we discovered that ubiquitylation is a key factor in the cellular response to high-LET radiation, including high-LET α-particles and protons. Specifically, we identified that histone H2B ubiquitylation on lysine 120, which is catalyzed by the E3 ubiquitin ligases ring finger protein 20/40 (RNF20/40) and male-specific lethal-2 homolog (MSL2), is induced following high-LET radiation. Induction of histone H2B ubiquitylation in turn promotes the repair of CDD required for cell survival (6). Subsequently, we utilized an siRNA screening strategy to identify that ubiquitin specific protease 6 (USP6) is important for maintaining levels of the DNA repair protein poly (ADP-ribose) polymerase-1 (PARP-1), which is similarly essential for efficient repair of CDD and thus survival of cells following irradiation with high-LET α-particles and protons (7). Our findings also highlighted the involvement of other DUBs in controlling cell survival under these conditions, although their precise roles and mechanisms of action in response to high-LET radiation are yet to be determined.

The ubiquitin-specific protease 9X (USP9X) is a highly conserved DUB, which has been suggested to play several important roles, particularly in maintaining cell survival, development, and polarity, as well as in protein trafficking (16). Specifically, USP9X has been shown to regulate apoptosis by initiating the apoptotic c-Jun N-terminal kinase (JNK) signaling cascade in neurons and embryonic kidney cells (17, 18). Conversely, USP9X is also proposed to be involved in the stabilization of the anti-apoptotic protein Mcl-1 by protecting it from ubiquitylation-dependent proteasomal degradation (19, 20), indicating that the role of USP9X in cell death regulation may be cell-type and disease specific. In fact, a role for USP9X in cancer development is not yet clear, having been found to act both as an oncogene and a tumor suppressor, depending on the type and stage of cancer (16). More recently, there has been accumulating evidence identifying USP9X as a major player in the maintenance and stability of key centriolar satellite proteins, consequently involved in promoting centrosome duplication (21–24). However, a role for USP9X in the cellular response to IR, and specifically the impact of the enzyme following high-LET radiation, has not been previously reported.

In this study, we have now further examined the roles of other DUBs that are required to promote cell survival specifically in response to high-LET radiation, with a focus on PBT that generates increasing LET particularly at the Bragg peak distal end. We demonstrate that an siRNA knockdown of USP9X leads to increased cell death specifically in response to high-LET protons and α-particle irradiation, but not to low-LET protons and X-rays. We further show that this reduction in cell survival in USP9X-depleted cells does not correlate with alterations in the efficiency of CDD repair or with the activation of cell death pathways, but is in fact mediated by centriolar satellite accumulation.

## Materials And Methods

### Antibodies and siRNA

The DUB siRNA library (ON-TARGETplus), USP9X siRNA pool and individual siRNA targeting USP9X (USP9X_6 5′-AGAAAUCGCUGGUAUAAAU-3′ and USP9X_8 5′-GUACGACGAUGUAUUCUCA-3′) were from Horizon Discovery (Cambridge, UK). The non-targeting control siRNA (AllStars Negative Control siRNA) was from Qiagen (Manchester, UK). The following antibodies were used: PARP-1 (sc-53643) and Mcl-1 (sc-819; both Santa Cruz Biotechnology, Heidelberg, Germany), γH2AX (05-636; Merck-Millipore, Watford, UK), USP9X (A301-350A) and 53BP1 (A300-272A; both Bethyl Labs, Montgomery, AL), LC3B (2775) and β-Galactosidase (9860; both Cell Signaling Technology, London, UK), Pericentrin (ab4448; Abcam, Cambridge, UK), CEP55 (23891-1-AP) and CEP131 (25735-1-AP; both Proteintech, Manchester, UK) and tubulin (T6199; Sigma-Aldrich, Gillingham, UK). Goat anti-mouse Alexa Fluor 555 (A21422) or goat anti-rabbit Alexa Fluor 488 (A11008) secondary antibodies for immunofluorescence were from Life Technologies (Paisley, UK).

### Cell Culture and Irradiation Sources

Oropharyngeal squamous cell carcinoma cells (UMSCC74A) were kindly provided by Prof T. Carey, University of Michigan, USA. HeLa and UMSCC74A cells were routinely cultured as monolayers in Dulbecco’s Modified Eagle Medium (DMEM) supplemented with 10% fetal bovine serum, 2 mM l-glutamine, 1× penicillin-streptomycin and 1× non-essential amino acids. siRNA knockdowns were performed for 48 h using Lipofectamine RNAiMAX (Life Technologies, Paisley, UK). Irradiation sources are as previously described (6). In brief, cells were exposed to either low-LET X-rays (100 kV; CellRad X-ray irradiator, Faxitron Bioptics, Tucson, USA; dose rate of ~3 Gy/min), or with a horizontal, passive-scattered proton beam line of 60 MeV maximal energy at the Clatterbridge Cancer Centre. These low-energy X-rays will generate higher LET photoelectrons and therefore a raised RBE when compared to higher energy photon sources (25, 26). For low-LET proton irradiations, cells were irradiated directly by a ~1 keV/µm pristine beam of 58 MeV effective energy (dose rate of ~5 Gy/min). For high-LET proton irradiations, a modulator was utilized to generate a 27-mm spread-out Bragg peak and a 24.4-mm absorber was used to position the cells at the distal edge, corresponding to a mean proton energy of 11 MeV at a dose averaged LET of 12 keV/µm (dose rate of ~5 Gy/min). The LET was an estimation based on previous work utilizing the Clatterbridge beam (27) and considering the proton stopping power at this range. RBE values of 1.67 ± 0.14 (Hela) and 1.85 ± 0.15 (UMSCC74A), comparing high-LET *versus* low-LET protons, have been previously determined (6). High-LET α-particle irradiations (3.26 MeV, 121 keV/µm) were performed on cells grown on Mylar sealed on glass cylinders using a ^238^Pu irradiator at the CRUK/MRC Oxford Institute for Radiation Oncology, as previously described (28, 29). Assuming a typical cross-sectional nuclear area of an attached Hela and UMSCC74A cell of ~150 µm^2^, these irradiations correspond to a mean of ~940, 78, and 7.7 nuclear traversals per Gy for low-LET protons, high-LET protons, and α-particle irradiations, respectively (29), with the number following a Poisson distribution and dependent on the natural variation in nuclear cross-sectional area with the cell cycle.

### Clonogenic Assays

Clonogenic assays were performed as recently described (6, 7). In brief, following siRNA treatments, cells were irradiated in 35-mm dishes, harvested and a defined number seeded in triplicate into 6-well plates. Plating efficiencies for the untreated cells were as follows: HeLa (~40%), UMSCC74A (~10%). Plating efficiencies for USP9X siRNA-treated cells were as follows: HeLa (~30%), UMSCC74A (~4%). Increasing cell numbers were plated for increasing IR doses to allow for reductions in plating efficiencies. Colonies were allowed to grow for 7 to 10 days prior to fixing and staining with 6% glutaraldehyde and 0.5% crystal violet for 30 min. Dishes were washed, left to air dry overnight and colonies counted using the GelCount colony analyzer (Oxford Optronics, Oxford, UK). Colony counting settings were optimized for both cell lines, based on inclusion of distinct colonies of specific size and intensity, although the same settings were used across the various treatments. A colony is defined as containing 50 or more cells. Relative colony formation (surviving fraction) was expressed as colonies per treatment level *versus* colonies that appeared in the untreated control. Results were accumulated from at least three independent biological experiments, apart from the siRNA screen which was from a single experiment (but containing triplicate samples).

### Cell Cycle Analysis and Immunoblotting

For cell cycle analysis, cells were trypsinized, washed twice with ice-cold PBS (100 × *g* for 5 min at 4°C), fixed with ice cold 70% ethanol and kept at 4°C until analysis. Fixed cells were centrifuged (200 × *g* for 5 min at 4°C), washed with PBS containing 0.05% Tween-20, and then resuspended in PBS containing 0.05% Tween-20, 10 µg/ml propidium iodide and 0.1 mg/ml RNase A for 1 h at room temperature. Analysis was performed using the Attune NxT Flow Cytometer (Life Technologies, Paisley, UK). Whole-cell extracts were prepared, separated by SDS-PAGE electrophoresis, and analyzed by quantitative immunoblotting using the Odyssey image analysis system (Li-cor Biosciences, Cambridge, UK), as previously described (30, 31).

### Enzyme-Modified Neutral Comet Assay

Detection of CDD was achieved using enzyme treatment of DNA originally described by Sutherland et al. (32, 33), but which has been employed in development of the enzyme-modified neutral comet assay, as recently described (34). In brief, cells were trypsinized, diluted to 1 × 10^5^ cells/ml and 250-µl aliquots of the cell suspension placed into the wells of a 24-well plate, which was placed on ice. Cells were irradiated (4 Gy) and embedded on a microscope slide in low melting agarose (Bio-Rad, Hemel Hempstead, UK). The slides were incubated for up to 4 h at 37°C in a humidified chamber to allow for DNA repair, prior to cell lysis in buffer containing 2.5 M NaCl, 100 mM EDTA, 10 mM Tris-HCl pH 10.5, 1% N-lauroylsarcosine, 1% DMSO, and 1% (v/v) Triton X-100. Slides were washed three times with enzyme reaction buffer (40 mM HEPES-KOH, 100 mM KCl, 0.5 mM EDTA and 0.2 mg/ml BSA, pH 8.0), and then incubated with either buffer alone (mock-treated; revealing levels of DNA DSBs) or with buffer containing 5 pmol OGG1, 6 pmol NTH1, and 0.6 pmol APE1 (enzyme-treated; revealing levels of DNA DSBs plus CDD) for 1 h at 37°C in a humidified chamber. Following treatment, slides were placed in cold electrophoresis buffer [1 × TBE buffer (pH 8.3)] in the dark for 25 min to allow the DNA to unwind, prior to electrophoresis at 25 V, ~20 mA for 25 min. Slides were washed three times with 1× PBS before allowing to dry overnight. Slides were rehydrated for 30 min in water (pH 8.0), stained for 30 min with SYBR Gold (Life Technologies, Paisley, UK), diluted 1:10,000 in water (pH 8.0), and again dried overnight. Cells (50 per slide, in duplicate) were analyzed from the dried slides using the Komet 6.0 image analysis software (Andor Technology, Belfast, Northern Ireland) and % tail DNA values averaged from at least three independent biological experiments.

### Immunofluorescence and β-Galactosidase Staining

Measurement of DNA repair protein foci (γH2AX and 53BP1) was examined as previously described (30). In brief, cells were grown on 13-mm coverslips until ~70% to 80% confluent, irradiated at 4 Gy and incubated for up to 8 h in 5% CO_2_ at 37°C to allow for DNA repair. Cells were washed with PBS at room temperature for 5 min, before being fixed using 4% paraformaldehyde for 10 min. Cells were permeabilized with 0.2% Triton X-100 in PBS for 10 min, washed three times with 0.1% Tween-20 for 10 min, and blocked to avoid non-specific staining *via* incubation with 2% BSA for 30 min at room temperature on a rocking platform. γH2AX or 53BP1 antibodies in 2% BSA were subsequently added and coverslips incubated overnight at 4°C. Following three washes with PBS, coverslips were incubated with either goat anti-mouse Alexa Fluor 555 or goat anti-rabbit Alexa Fluor 488 secondary antibodies in 2% BSA for 1 h at room temperature in the dark. Finally, samples were washed with PBS for 10 min on a rocking platform and mounted on a microscope slide using Fluoroshield containing DAPI (Sigma-Aldrich, Gillingham, UK). Cells were examined using an Olympus BX61 fluorescent microscope with a Photometrics CoolSNAP HQ2 CCD camera. MicroManager software was used to capture images (~20 images/cell line/antibody). Similarly, cells were fixed on coverslips post-irradiation (4 Gy) and incubation (48 h), but then stained using Pericentrin antibodies prior to incubation with AlexaFluor488 secondary antibodies, and pericentrin-rich foci scored using fluorescence microscopy. Analysis of senescence was carried out using the β-Galactosidase Staining kit (Cell Signaling Technology, London, UK) and where hydrogen peroxide (100 µM for 2 h) was used as a positive control.

### Statistical Analysis

All experiments (unless defined above) were performed in at least triplicate as separate independent, biological experiments. Statistical analysis of clonogenic survival data was performed using a one-way ANOVA and comparing surviving fractions at 2 Gy (SF2) or 4 Gy (SF4). Statistical analysis of DNA damage quantified through neutral comet assays and pericentrin-rich foci through immunofluorescence staining was performed using either a one-sample or two-sample *t* test.

## Results

### Screening for DUBs Involved in Response to High-LET IR

**Figure 1:**
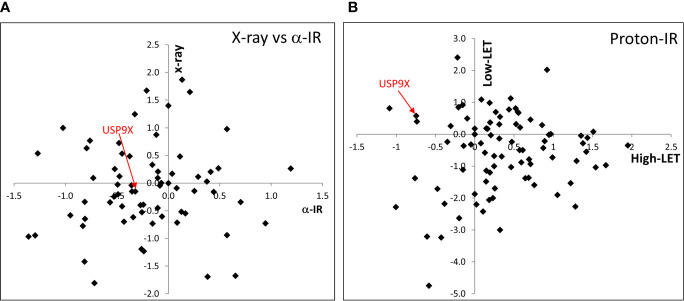
siRNA screen of DUBs modulating cell survival following high and low LET radiation. HeLa cells were transfected with siRNA (pool of 4 oligonucleotides) targeting individual DUBs for 48 h, and irradiated with either **(A)** 0.5 Gy α-particles or 1 Gy X-rays, or **(B)** 2 Gy relatively high-LET protons or 2 Gy low-LET protons. Clonogenic survival was analyzed from a single experiment (using triplicate samples) and normalized against the mock-treated control. Results are shown as a log_2_ plot, and indicated is the clonogenic survival following USP9X depletion. Data has been adapted from (7).

In our recent work, we performed an siRNA screen of 84 DUBs analyzing the survival of HeLa cells after a single dose of IR, with a particular focus on high-LET IR, which generates increased levels and complexity of CDD consisting of two or more DNA lesions within one or two helical turns of the DNA (7). We have now further interrogated these data, which demonstrates the wide range of changes in HeLa cell survival in response to a single dose of high-LET α-particles (121 keV/µm) *versus* low-LET X-rays following individual DUB knockdowns (**Figure 1A**). Similarly, a variability in cell survival in the absence of the DUBs is observed in response to relatively high-LET protons (12 keV/µm) generated at the Bragg peak distal edge, *versus* low-LET protons (1 keV/µm) at the entrance dose of a pristine beam (**Figure 1B**). Survival was normalized to the irradiated mock-treated control sample (generating ~40% cell survival), which was set to 1.0, and the data subsequently plotted (on a log_2_ scale). The top 10 DUB candidates reducing cell survival in response to high-LET α-particles (**Table 1**) and relatively high-LET protons (**Table 2**), in addition to their impact on survival following low-LET protons and X-rays, are shown. We focused our attention on candidate enzymes whose depletion led to a specific reduction in cell survival following high-LET radiation. This led us to identify that depletion of USP9X caused no reduction in cell survival after low-LET X-ray or proton irradiation, whereas survival after high-LET α-particles and protons was reduced by ~20% and ~40%, respectively (**Tables 1**, **2**).

**Table 1:** Candidate DUBs whose siRNA-mediated depletion enhance radiosensitivity of cells in response to α-particle radiation.

DUB enzyme	α-particles	X-rays	High-LET protons	Low-LET protons
USP6	0.39	0.51^*^	0.60	1.32
USP21	0.41	0.52	1.06	0.82
USP36	0.41	1.45	0.66	0.11
DUB3	0.49	2.00	0.90	1.89
STAMBPL1	0.52	0.67	0.72	0.31
UCHL3	0.56	0.59	1.69	0.33
AMSH	0.57	0.37	1.47	1.59
CYLD	0.57	0.79	0.47	1.76
UCHL5	0.57	0.64	1.51	0.54
OTUB1	0.58	1.55	1.99	0.61
**USP9X**	**0.80**	**0.90**	**0.60**	**1.50**

Surviving fractions from a single experiment containing triplicate samples were normalized against the mock-treated control, which was set to 1.0. Survival data acquired in response to USP9X depletion is highlighted in bold. ^*^Previously identified as a false positive result, data adapted from (7).

**Table 2:** Candidate DUBs whose siRNA-mediated depletion enhance radiosensitivity of cells in response to high-LET protons.

DUB enzyme	High-LET protons	Low-LET protons	α-particles	X-rays
CYLD	0.47	1.76	0.57	0.79
USP7	0.50	0.21	1.30	1.02
USP31	0.59	0.38	n.d.	n.d.
**USP9X**	**0.60**	**1.50**	**0.80**	**0.90**
USP6	0.60	1.32	0.39	0.51^*^
USP36	0.66	0.11	0.41	1.45
USP39	0.67	0.04	n.d.	2.64
STAMBPL1	0.72	0.31	0.52	0.67
USP43	0.74	0.11	n.d.	n.d.
YOD1	0.77	0.22	0.78	0.90

Surviving fractions from a single experiment containing triplicate samples were normalized against the mock-treated control, which was set to 1.0. n.d. indicates not determined. Survival data acquired in response to USP9X depletion is highlighted in bold. ^*^Previously identified as a false positive result, data adapted from (7).

### USP9X Modulates Cell Survival in Response to High-LET IR

**Figure 2:**
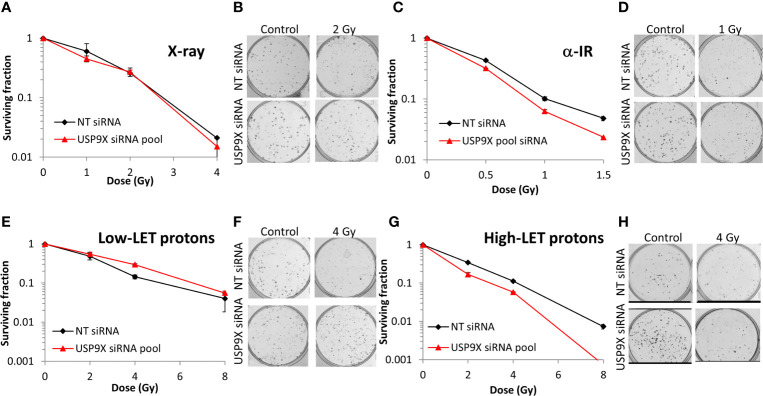
Validation of USP9X in enhancing cellular radiosensitivity in response to high-LET α-particles and protons. HeLa cells were treated with USP9X siRNA (pool of 4 oligonucleotides) or a non-targeting (NT) control siRNA for 48 h and irradiated with increasing doses of **(A, B)** X-rays, **(C, D)** α-particles, **(E, F)** low-LET protons, or **(G, H)** relatively high-LET protons. Clonogenic survival of cells was analyzed from 2 independent experiments, and shown is the mean surviving fraction ± S.E. (**B, D, F, H**) Shown are representative images of colonies in non-irradiated and irradiated plates, the latter of which contained four times the number of cells seeded.

**Figure 3:**
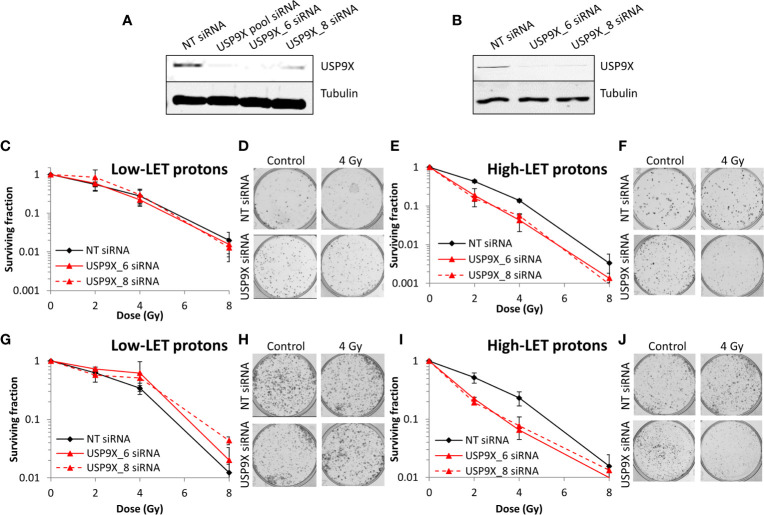
Specific targeting of USP9X leads to enhanced cellular radiosensitivity after relatively high-LET protons. **(A–J)** Cells were treated with individual USP9X siRNAs (USP9X_6 and USP9X_8) or a non-targeting (NT) control siRNA for 48 h. Whole cell extracts prepared from **(A)** HeLa or **(B)** UMSCC74A cells were analyzed by immunoblotting using USP9X or tubulin antibodies. HeLa cells were subsequently irradiated with increasing doses of **(C, D)** low-LET protons or **(E, F)** relatively high-LET protons. UMSCC74A cells were also treated with increasing doses of **(G, H)** low-LET protons or **(I, J)** relatively high-LET protons. Clonogenic survival of cells was analyzed from 3 independent experiments, and shown is the mean surviving fraction ± S.E. Shown are representative images of colonies from **(D, F)** HeLa cells or **(H, J)** UMSCC74A cells in non-irradiated and irradiated plates, the latter of which contained four times the number of cells seeded. A comparison of the surviving fractions at 2 Gy (SF2) by one-way ANOVA in HeLa cells reveals p < 0.05 (NT siRNA *vs* USP9X_6), p < 0.001 (NT siRNA *vs* USP9X_8) and in UMSCC74A cells, p < 0.05 (NT siRNA *vs* USP9X_6 and NT siRNA *vs* USP9X_8). At 4 Gy (SF4), one-way ANOVA values in HeLa cells are p < 0.01 (NT siRNA *vs* USP9X_6), p < 0.005 (NT siRNA *vs* USP9X_8) and in UMSCC74A cells, p < 0.05 (NT siRNA *vs* USP9X_6 and NT siRNA *vs* USP9X_8).

Following siRNA screening, we aimed to validate that depletion of USP9X caused a decrease in cell survival following high-LET radiation, but had no impact in response to low-LET protons, in both HeLa cells and also cells derived from head and neck squamous cell carcinoma. In response to USP9X siRNA (pool of 4 siRNAs utilized in the screen), there was no impact on cell survival following a dose titration of low-LET X-rays (**Figures 2A, B**) or low-LET protons (**Figures 2E, F**) in comparison to non-targeting (NT) control siRNA-treated cells. In fact, depletion of USP9X appeared to cause a mild increase in radioresistance following low-LET protons. However, in contrast to low-LET radiation, USP9X siRNA led to reduced survival in response to high-LET α-particles (**Figures 2C, D**), and more dramatically in response to relatively high-LET protons (**Figures 2G, H**). To further validate that our observations were specific, we utilized two single siRNA sequences (USP9X_6 and USP9X_8) that were demonstrated to be effective in suppressing USP9X protein levels in both HeLa (**Figure 3A**) and UMSCC74A oropharyngeal squamous cell carcinoma cells (**Figure 3B**). We focused on the impact of protons, particularly as PBT is employed clinically for the treatment of tumors, including head and neck squamous cell carcinoma. We confirmed that targeting USP9X had no effect on the survival of HeLa cells (**Figures 3C, D**) or UMSCC74 cells (**Figures 3G, H**) following low-LET protons, in comparison to NT control siRNA-treated cells. However, significantly reduced survival of HeLa (**Figures 3E, F**) and UMSCC74A (**Figures 3I, J**) cells was observed following USP9X depletion in response to high-LET protons, compared with NT control siRNA cells. These data confirmed the importance of USP9X in maintaining cell survival following treatment with relatively high-LET protons.

### USP9X Does Not Interfere With CDD Repair or Cell Cycle Progression Following High-LET Protons

**Figure 4:**
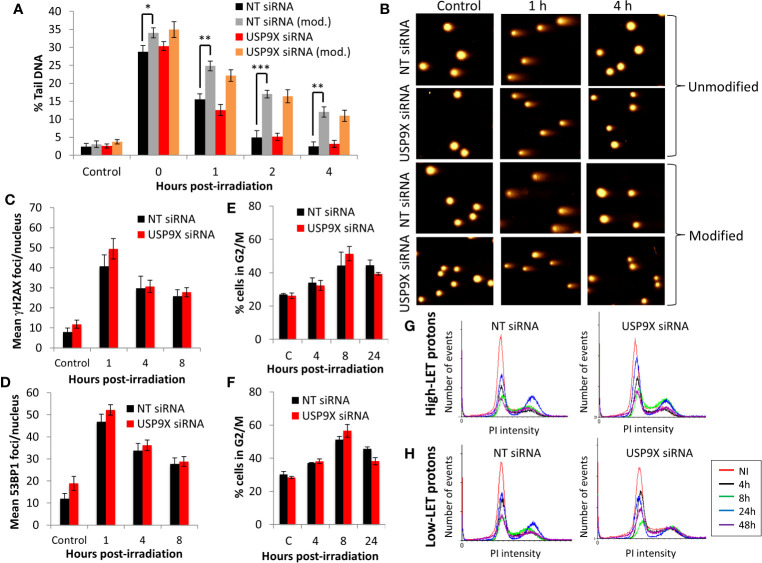
USP9X depletion does not affect the repair of CDD or cell cycle progression in response to relatively high-LET protons. HeLa cells were treated with USP9X or a non-targeting (NT) control siRNA for 48 h. **(A)** Cells were irradiated with 4 Gy relatively high-LET protons, and DNA damage measured at various time points post-irradiation using the enzyme-modified neutral comet assay in the absence (revealing DSBs) or presence (revealing CDD; as indicated by mod) of the recombinant enzymes APE1, NTH1, and OGG1. Shown is the mean percentage tail DNA ± S.D plus **(B)** representative images of comets. *p < 0.02, **p < 0.005, ***p < 0.002 as analyzed by a one-sample *t*-test. **(C)** γH2AX or **(D)** 53BP1 foci were analyzed by immunofluorescence staining at various time points post-irradiation. Shown is the mean number of foci/nucleus ± S.D. **(E–H)** Cell cycle profiles were determined by flow cytometry analysis post-irradiation with **(E, G)** high-LET or **(F, H)** low-LET protons. C refers to control, unirradiated cells. **(E, F)** Shown is the mean percentage of cells in G2/M phase ± S.E.

Given our demonstration that USP9X is required for maintaining cell survival specifically in response to relatively high-LET protons, we sought to define its mechanism of action. In the first instance, and given the propensity for high-LET radiation to cause increases in the levels of CDD, we utilized an enzyme-modified neutral comet assay (34) to monitor the levels and kinetics of repair of CDD. This assay also has the added advantage of revealing the levels and repair of DNA DSBs (in the absence of modifying enzymes). We demonstrate that DNA DSB levels are not significantly different in USP9X siRNA *versus* NT control siRNA-treated cells in response to relatively high-LET protons at any time points (0-4 h) post-irradiation (**Figure 4A**; compare black and red, and **Figure 4B**). We were able to confirm that relatively high-LET protons caused a significant increase in the levels of CDD in NT control siRNA-treated cells immediately post-irradiation, followed by significantly slower kinetics of repair from 1 to 4 h post-irradiation (**Figure 4A**; compare black and grey bars, and **Figure 4B**), consistent with our previously published data (6, 7). Following depletion of USP9X, we observed that the efficiency of CDD repair in comparison to NT control siRNA-treated cells was not significantly different, at least at 1 to 4 h post-irradiation (**Figure 4A**; compare grey and orange bars, and **Figure 4B**). Data, demonstrating a lack of effect of USP9X depletion on the repair of DNA DSBs induced by relatively high-LET protons, were supported by an absence of any differences in the induction and resolution of γH2AX and 53BP1 foci, as surrogate markers, up to 8 h post-irradiation relative to NT control siRNA-treated cells (**Figures 4C, D** **)**. We also analyzed the progression of cells through the cell cycle post-irradiation. Similarly, this revealed no significant differences in G2/M accumulation in USP9X siRNA-treated cells either before or after high-LET proton irradiation compared to NT control siRNA-treated cells (**Figure 4E**), or in the overall cell cycle profiles (**Figure 4G**). Similar numbers of cells in G2/M phase (**Figure 4F**) and in cell cycle profiles (**Figure 4H**) were also observed in USP9X-depleted and NT control siRNA-treated cells in response to low-LET protons. These data suggest that there is no significantly different checkpoint activation or cell cycle arrest in USP9X-depleted cells following either relatively high- or low-LET protons.

### USP9X Does Not Impact Apoptosis, Autophagy or Senescence Following High-LET Protons

**Figure 5:**
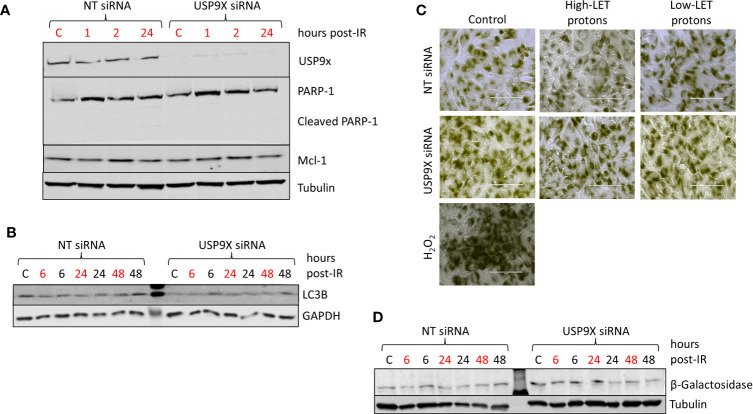
USP9X depletion does not affect cellular levels of apoptosis, autophagy and senescence in response to relatively high-LET protons. HeLa cells were treated with USP9X or a non-targeting (NT) control siRNA for 48 h, and unirradiated (designated C) or irradiated with 4 Gy relatively high-LET or low-LET protons and allowed to repair for the time points indicated post-irradiation. **(A, B, D)** Whole cell extracts were analyzed by immunoblotting using the indicated antibodies. **(C)** Cells were fixed 48 h post-irradiation and stained for β-Galactosidase, with 100 µM H_2_O_2_ used as a positive control.

To examine the potential impact of USP9X on triggering apoptosis post-irradiation with relatively high-LET protons, we analyzed the cellular protein levels of PARP-1 (including cleaved PARP-1) and Mcl-1. However, we did not observe any differences in these proteins 1 to 24 h post-irradiation, nor in the accumulation of cleaved PARP-1, in USP9X siRNA-treated cells compared to NT control siRNA-treated cells (**Figure 5A**), suggesting that apoptosis does not play a role in the differential cellular response. We also compared the expression of LC3B (**Figure 5B**; red text refers to time following relatively high-LET protons) and β-Galactosidase by both microscopy (**Figure 5C**; utilizing H2O2 as a positive control) and immunoblotting (**Figure 5D**), as markers of autophagy and senescence, respectively. Similarly, we observed no obvious differences in LC3B or β-galactosidase levels between USP9X-depleted and NT control siRNA-treated cells post-irradiation with either relatively high- or low-LET protons, suggesting that neither autophagy nor senescence are responsible for decreased cell survival specifically following relatively high-LET protons.

### USP9X Inhibition Causes Amplification of Pericentrin-Rich Bodies After High-LET Protons

**Figure 6:**
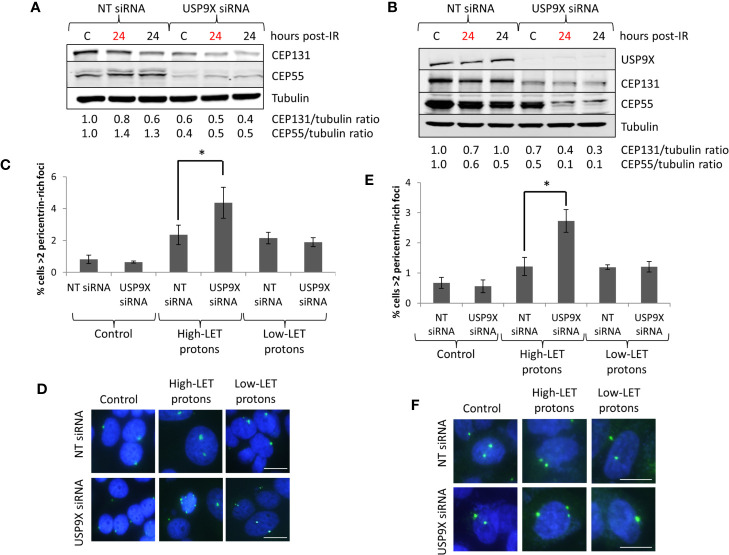
USP9X depletion destabilizes centrosomal proteins, and induces pericentrin-rich foci amplification in response to relatively high-LET protons. **(A, C, D)** HeLa or **(B, E, F)** UMSCC74A cells were treated with USP9X or a non-targeting (NT) control siRNA for 48 h, and unirradiated designated **(C)** or irradiated with 4 Gy relatively high-LET or low-LET protons and allowed to repair for the time points indicated post-irradiation. **(A, B)** Whole cell extracts were analyzed by immunoblotting using the indicated antibodies. Red and black text refers to time post-irradiation following relatively high-LET and low-LET protons, respectively. Protein levels of CEP131 and CEP55 relative to tubulin and normalized to the unirradiated NT siRNA control treated cells which was set to 1.0 are shown. **(C, E)** Analysis of pericentrin was performed by immunofluorescence 48 h post-irradiation. Shown is the mean percentage of cells with >2 pericentrin-rich foci/nucleus ± S.D. *p < 0.03 as analyzed by a two-sample *t*-test. **(D, F)** Representative images of pericentrin staining (green stain) and nuclei (blue stain; DAPI).

Since recent data have shown that USP9X is an integral component of centrosomal protein maintenance, we analyzed the impact of USP9X depletion on stabilization of centrosomal proteins. We observed that an absence of USP9X alone in HeLa cells caused reduced levels of the centrosome proteins CEP55 (~60%) and CEP131 (~40%) in comparison to NT control siRNA-treated cells (**Figure 6A**; compare lanes 1 and 4). Reduced levels of CEP55 and CEP131 (by ~50–60%) were maintained in USP9X-depleted cells 24 h post-irradiation with both low- and high-LET protons (**Figure 6A**; compare lanes 2–3 and 5–6). Similar observations were seen in UMSCC74A cells, whereby CEP55 and CEP131 protein levels were reduced by 50% and 30%, respectively, in USP9X-depleted cells (**Figure 6B**; compare lanes 1 and 4). CEP55 and CEP131 protein levels were further reduced (by ~60–90%) in USP9X-depleted cells compared to NT control siRNA-treated cells 24 h post-irradiation with both low- and high-LET protons (**Figure 6B**; compare lanes 2–3 and 5–6). In addition to reduced centrosome protein stability, we discovered a ~2.6- to 2.9-fold increase in pericentrin-rich foci in NT control siRNA-treated HeLa cells 48 h post-irradiation with both low and high LET protons (**Figures 6C, D** **)**. However, we observed that there was an additional ~1.9-fold statistically significant increase in foci in USP9X-depleted cells specifically in response to high-LET protons, but not following low-LET protons (**Figures 6C, D** **)**. Similarly, in UMSCC74A cells, there was a ~1.8-fold increase in pericentrin-rich foci in NT control siRNA-treated HeLa cells 48 h post-irradiation with both low- and high-LET protons, but this further increased by ~2.2-fold in USP9X-depleted cells (**Figures 6E, F** **)**. Amplification of pericentrin-foci was consequently ~6.7-fold (HeLa) and ~4.9-fold (UMSCC74A) higher in USP9X-depleted cells irradiated with high-LET protons compared with unirradiated cells.

**Figure 7:**
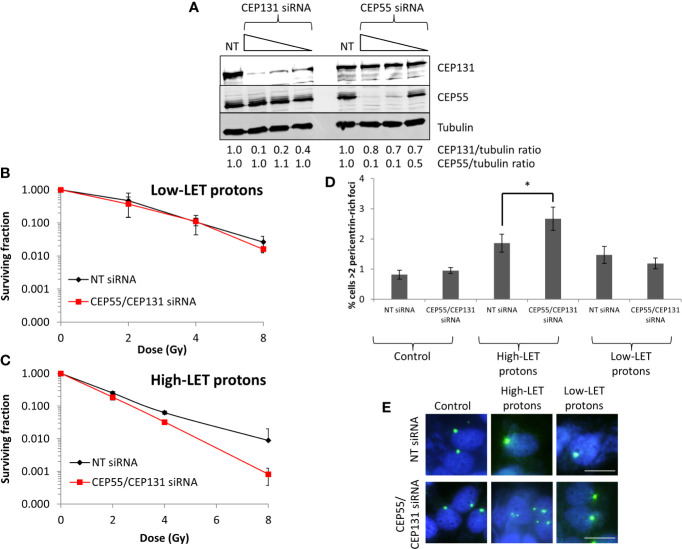
CEP55 and CEP131 depletion induces pericentrin-rich foci amplification and reduces cell survival in response to relatively high-LET protons. **(A)** HeLa cells were treated with CEP55 or CEP131 siRNA (0.01, 0.1 and 1 nM) or a non-targeting (NT) control siRNA for 48 h, and whole cell extracts were analyzed by immunoblotting using the indicated antibodies. Protein levels of CEP131 and CEP55 relative to tubulin and normalized to the NT siRNA control-treated cells which was set to 1.0 are shown. **(B–E)** HeLa cells were treated with CEP55 and CEP131 siRNA (0.01 nM) or a non-targeting (NT) control siRNA for 48 h. Cells were irradiated with increasing doses of **(B)** low-LET protons or **(C)** relatively high-LET protons. Clonogenic survival of cells was analyzed from 2 independent experiments, and shown is the mean surviving fraction ± S.E. **(D, E)** Alternatively, analysis of pericentrin was performed by immunofluorescence 48 h post-irradiation (with 4 Gy). **(D)** Shown is the mean percentage of cells with >2 pericentrin-rich foci/nucleus ± S.D. *p < 0.05 as analyzed by a two-sample *t*-test. **(E)** Representative images of pericentrin staining (green stain) and nuclei (blue stain; DAPI).

To recapitulate the impact of USP9X in controlling sensitivity of cells to high-LET protons mediated *via* centrosomal protein maintenance, we specifically targeted CEP55 and CEP131 using siRNA. We identified conditions that led to ~50% reduced CEP55 and CEP131 protein levels in HeLa cells (**Figure 7A**; lanes 4 and 8), which mimic those of USP9X depletion (**Figure 6A**). We observed under these conditions that cells do not display any increase in radiosensitivity to low-LET protons (**Figure 7B**), but that there is reduced survival to relatively high-LET protons (**Figure 7C**). Additionally, we discovered that there was an a ~1.4-fold statistically significant increase in pericentrin-rich foci in USP9X-depleted cells *versus* NT control siRNA-treated cells specifically in response to high-LET protons, but not following low-LET protons (**Figures 7D, E** **)**. This suggests that USP9X is required for maintenance of centrosomal proteins and for controlling pericentrin-rich foci, which is important for cell survival particularly in response to high-LET radiation.

## Discussion

IR, particularly at high-LET, can generate CDD known to compromise genome integrity and to contribute to cell death due to the inability of the cellular DNA repair machinery to accurately repair the damage. However, high-LET radiation has also been shown to promote persistent alterations to the structure of chromatin (9, 10), and to enhance senescence and apoptosis (11, 12). We recently demonstrated using a DUB siRNA screen, that depletion of USP6 causes significantly increased radiosensitivity to high-LET IR (relatively high-LET protons and α-particles) but not low-LET IR (protons or X-rays), which was mediated by instability of PARP-1 required for CDD repair (7). In the current study, we discovered that another DUB enzyme, USP9X, is similarly required for maintaining cell survival specifically in response to high-LET IR, particularly high-LET protons. We demonstrated that this phenotype was not caused by interference with CDD repair, cell cycle progression, apoptosis, autophagy or senescence. In fact, we discovered that an absence of USP9X caused destabilization of centrosomal proteins, and enhanced amplification of pericentrin-rich foci in response to relatively high-LET protons. This suggests that maintenance of CEP55 and CEP131 is the major factor contributing to reduced cell survival following relatively high-LET protons in the absence of USP9X.

Our findings that USP9X does not affect cell cycle progression is in agreement with a previous study, which demonstrated that USP9X depletion was linked to an increased loss of anaphase-promoting complex/cyclosome (APC/C) substrates, in particular cyclin A, but also cyclin B and NEK2A, and that cells consequently failed to arrest mitosis after microtubule poisoning in HeLa cells and U2OS cells (35). USP9X has been shown to promote Mcl-1 stabilization and to increase tumor cell survival in response to radiation and chemotherapy in several tumor types (19, 36–39). This is in contrast to our findings, where we observed no evidence of a role for Mcl-1, and therefore apoptosis through lack of PARP-1 cleavage, in the USP9X-dependent radiosensitisation of HeLa and UMSCC74A cells following relatively high-LET protons. Indeed, in certain glioblastoma cell lines, it has been demonstrated that USP9X loss regulates radiosensitivity by Mcl-1-independent mechanisms (40). USP9X interacts with VMP1, indicating that there is a close cooperation between the autophagy pathway and the ubiquitin recognition machinery required for selective autophagosome formation (41). On analysis of the levels of LC3B, as a marker of autophagy, after relatively high-LET *versus* low-LET protons, we did not find any evidence indicating that autophagy plays a significant role in cell survival in response to these conditions. USP9X has recently been suggested to stabilize the breast cancer protein-1 (BRCA1) required for DSB repair by homologous recombination through its DUB enzymatic activity, and which is required for resistance to the PARP inhibitor olaparib in HeLa and breast cancer cells (42). These data are supported by another study, although it was proposed that USP9X actually regulates BRCA1 (and RAD51) at the transcriptional level in U2OS cells, which was independent of its catalytic activity (43). This study also presented limited data showing increased levels of 53BP1 foci both in the absence and presence of IR (1 h post-irradiation), in USP9X-depleted cells. Despite this evidence, we observed that an absence of USP9X did not cause any significant differences in the repair of DSBs, using γH2AX and 53BP1 foci analysis, or in the kinetics of DSB or CDD repair after relatively high-LET protons. This would indicate that the ability of the cells, at least the ones utilized in our study (HeLa and UMSCC74A), to repair DNA damage was not heavily compromised.

USP9X has more recently been observed in U2OS and HeLa cells to localize to centrosomes in association with PCM1, CEP55 and CEP131, where the enzyme antagonizes proteosomal degradation of these key centriolar satellite proteins to promote centrosome duplication (21–23). Interestingly, in MRXS99F fibroblasts USP9X was also found to localize to the centrosome, but had no impact on controlling the protein levels of PCM1 or CEP131 (24). This suggests that there are cell-type–specific roles and targets for USP9X. Here, we observed that USP9X-depleted HeLa and UMSCC74A oropharyngeal squamous cell carcinoma cells have reduced levels of CEP55 and CEP131. Furthermore, we observed an increased number of pericentrin-rich foci after proton irradiation in both non-targeting and USP9X-depleted cells, but that this phenotype was further exacerbated specifically after high-LET protons in USP9X-depleted cells. We also targeted CEP55 and CEP131 directly and demonstrated that their moderate depletion (by ~50%, consistent with effects seen following USP9X depletion) also led to amplification of pericentrin-rich foci and enhanced sensitivity of cells specifically to high LET protons. Pericentrin is not only a component of centrosomes but also a feature of centriolar satellites and cilia, which are associated with centrosome biology. Our data therefore indicate that USP9X could play a role in centriolar satellite generation, which ultimately maintains centrosome stability. Centriolar satellites are small, granular structures that cluster around centrosomes and are thought to play important roles in both centrosome assembly and in cilia formation (44, 45). There is also evidence demonstrating that cellular stresses, such as UV radiation, disrupt centriolar satellites and stimulate ciliogenesis (46). Furthermore, and consistent with our data, DNA damage induction by IR and bleomycin has been shown to lead to excessive formation of centriolar satellites, which is a prerequisite for centrosome amplification (47). It is possible, therefore, that following high-LET protons, which is more densely ionizing leading to increased protein as well as DNA damage, there is uncontrolled centrosome biogenesis, which is likely to contribute to increased genome instability through chromosomal aberrations and therefore reduced cell survival. It is important to note that the UMSCC74A cells employed in our study contain wild type p53 tumor suppressor protein, whereas HeLa cells have significantly reduced levels of p53 due to human papillomavirus type 18 infection that ultimately leads to p53 degradation. The phenotype of increased radiosensitivity and elevated levels of pericentrin-rich foci in cells in response to high-LET radiation therefore does not appear to be dependent on loss of p53. Given that p53 is frequently mutated in human cancers, and particularly in head and neck squamous cell carcinoma (48), more expansive studies using cells containing different p53 status are required to fully investigate this. Nevertheless, and at this stage, the precise mechanism underlying the impact of USP9X in regulating centrosome biology specifically in response to relatively high-LET protons is unclear. Further ongoing experiments are necessary to fully understand centrosome biology (>100 centrosome proteins exist) in multiple cell lines lacking USP9X pre- and post-irradiation, particularly those originating from head and neck cancers, which is our research and clinical focus. We also aim to analyze more the impact of low- *versus* high-LET radiation at the chromosome level, and the dependence on USP9X, which is likely to be contributing to the increased radiosensitivity of cells lacking USP9X specifically in response high-LET radiation. Nevertheless, our data suggest that USP9X is essential for maintaining cell survival, particularly following high-LET radiation.

## Data Availability Statement

The raw data supporting the conclusions of this article will be made available by the authors, without undue reservation.

## Author Contributions

JP conceptualized and designed the project. AK, MH, and JP designed the experimental setup. CN, MF, RC, and JH performed experiments. CN, MF, RC, and JP performed data analysis and validation. CN, MF, RC, and JP wrote the manuscript and all authors contributed to reviewing and editing. JP and MH coordinated funding acquisition. All authors contributed to the article and approved the submitted version.

## Funding

This research was funded by North West Cancer Research (CR1074 and CR1197) and by the Medical Research Council *via* a New Investigator Research Grant (MR/M000354/1) awarded to JP. Funding from Medical Research Council Strategic Partnership Funding (MC-PC-12004) for the CRUK/MRC Oxford Institute for Radiation Oncology is also gratefully acknowledged.

## Conflict of Interest

The authors declare that the research was conducted in the absence of any commercial or financial relationships that could be construed as a potential conflict of interest.

## References

[1] Holliday EB Frank SJ. Proton Radiation Therapy for Head and Neck Cancer: A Review of the Clinical Experience to Date. Int J Radiat Oncol Biol Phys (2014) 89:292–302. 10.1016/j.ijrobp.2014.02.029 24837890

[2] Goodhead DT. Initial Events in the Cellular Effects of Ionizing Radiations: Clustered Damage in DNA. Int J Radiat Biol (1994) 65:7–17. 10.1080/09553009414550021 7905912

[3] Ward JF. The Complexity of DNA Damage: Relevance to Biological Consequences. Int J Radiat Biol (1994) 66:427–32. 10.1080/09553009414551401 7983426

[4] Hill MA. Radiation Track Structure: How the Spatial Distribution of Energy Deposition Drives Biological Response. Clin Oncol (R Coll Radiol) (2020) 32:75–83. 10.1016/j.clon.2019.08.006 31511190

[5] Vitti ET Parsons JL. The Radiobiological Effects of Proton Beam Therapy: Impact on DNA Damage and Repair. Cancers (Basel) (2019) 11:946. 10.3390/cancers11070946 PMC6679138 31284432

[6] Carter RJ Nickson CM Thompson JM Kacperek A Hill MA Parsons JL. Complex DNA Damage Induced by High Linear Energy Transfer Alpha-Particles and Protons Triggers a Specific Cellular DNA Damage Response. Int J Radiat Oncol Biol Phys (2018) 100:776–84. 10.1016/j.ijrobp.2017.11.012 PMC5796827 29413288

[7] Carter RJ Nickson CM Thompson JM Kacperek A Hill MA Parsons JL. Characterisation of Deubiquitylating Enzymes in the Cellular Response to High-LET Ionizing Radiation and Complex Dna Damage. Int J Radiat Oncol Biol Phys (2019) 104:656–65. 10.1016/j.ijrobp.2019.02.053 PMC6542414 30851349

[8] Mavragani IV Nikitaki Z Souli MP Aziz A Nowsheen S Aziz K. Complex DNA Damage: A Route to Radiation-Induced Genomic Instability and Carcinogenesis. Cancers (Basel) (2017) 9:91. 10.3390/cancers9070091 PMC5532627 28718816

[9] Timm S Lorat Y Jakob B Taucher-Scholz G Rube CE. Clustered DNA Damage Concentrated in Particle Trajectories Causes Persistent Large-Scale Rearrangements in Chromatin Architecture. Radiother Oncol (2018) 129:600–10. 10.1016/j.radonc.2018.07.003 30049456

[10] Anderson RM Stevens DL Sumption ND Townsend KM Goodhead DT Hill MA. Effect of Linear Energy Transfer (LET) on the Complexity of Alpha-Particle-Induced Chromosome Aberrations in Human CD34+ Cells. Radiat Res (2007) 167:541–50. 10.1667/RR0813.1 17474795

[11] Zhang X Ye C Sun F Wei W Hu B Wang J. Both Complexity and Location of DNA Damage Contribute to Cellular Senescence Induced by Ionizing Radiation. PloS One (2016) 11:e0155725. 10.1371/journal.pone.0155725 27187621 PMC4871470

[12] Niemantsverdriet M Van Goethem MJ Bron R Hogewerf W Brandenburg S Langendijk JA. High and Low LET Radiation Differentially Induce Normal Tissue Damage Signals. Int J Radiat Oncol Biol Phys (2012) 83:1291–7. 10.1016/j.ijrobp.2011.09.057 22245200

[13] Edmonds MJ Parsons JL. Regulation of Base Excision Repair Proteins by Ubiquitylation. Exp Cell Res (2014) 329:132–8. 10.1016/j.yexcr.2014.07.031 25108137

[14] Carter RJ Parsons JL. Base Excision Repair, a Pathway Regulated by Posttranslational Modifications. Mol Cell Biol (2016) 36:1426–37. 10.1128/MCB.00030-16 PMC4859697 26976642

[15] Dianov GL Meisenberg C Parsons JL. Regulation of DNA Repair by Ubiquitylation. Biochem (Mosc) (2011) 76:69–79. 10.1134/S0006297911010093 21568841

[16] Murtaza M Jolly LA Gecz J Wood SA. La FAM Fatale: USP9X in Development and Disease. Cell Mol Life Sci (2015) 72:2075–89. 10.1007/s00018-015-1851-0 PMC4427618 25672900

[17] Huntwork-Rodriguez S Wang B Watkins T Ghosh AS Pozniak CD Bustos D. JNK-Mediated Phosphorylation of DLK Suppresses its Ubiquitination to Promote Neuronal Apoptosis. J Cell Biol (2013) 202:747–63. 10.1083/jcb.201303066 PMC3760612 23979718

[18] Nagai H Noguchi T Homma K Katagiri K Takeda K Matsuzawa A. Ubiquitin-Like Sequence in ASK1 Plays Critical Roles in the Recognition and Stabilization by USP9X and Oxidative Stress-Induced Cell Death. Mol Cell (2009) 36:805–18. 10.1016/j.molcel.2009.10.016 20005844

[19] Schwickart M Huang X Lill JR Liu J Ferrando R French DM. Deubiquitinase USP9X Stabilizes MCL1 and Promotes Tumour Cell Survival. Nature (2010) 463:103–7. 10.1038/nature08646 20023629

[20] Sun H Kapuria V Peterson LF Fang D Bornmann WG Bartholomeusz G. Bcr-Abl Ubiquitination and Usp9x Inhibition Block Kinase Signaling and Promote CML Cell Apoptosis. Blood (2011) 117:3151–62. 10.1182/blood-2010-03-276477 21248063

[21] Li X Song N Liu L Liu X Ding X Song X. USP9X Regulates Centrosome Duplication and Promotes Breast Carcinogenesis. Nat Commun (2017) 8:14866. 10.1038/ncomms14866 28361952 PMC5380967

[22] Wang Q Tang Y Xu Y Xu S Jiang Y Dong Q. The X-linked Deubiquitinase USP9X Is an Integral Component of Centrosome. J Biol Chem (2017) 292:12874–84. 10.1074/jbc.M116.769943 PMC5546028 28620049

[23] Han KJ Wu Z Pearson CG Peng J Song K Liu CW. Deubiquitylase USP9X Maintains Centriolar Satellite Integrity by Stabilizing Pericentriolar Material 1 Protein. J Cell Sci (2019) 132:jcs221663. 10.1242/jcs.221663 30584065 PMC6362394

[24] Kodani A Moyer T Chen A Holland A Walsh CA Reiter JF. SFI1 Promotes Centriole Duplication by Recruiting USP9X to Stabilize the Microcephaly Protein STIL. J Cell Biol (2019) 218:2185–97. 10.1083/jcb.201803041 PMC6605807 31197030

[25] Hill MA. The Variation in Biological Effectiveness of X-Rays and Gamma Rays With Energy. Radiat Prot Dosimetry (2004) 112:471–81. 10.1093/rpd/nch091 15623881

[26] Task Group on Radiation Quality Effects in Radiological Protection C.O.R.E I.C.O.R.P. Relative Biological Effectiveness (RBE), Quality Factor (Q), and Radiation Weighting Factor (W(R)). A Report of the International Commission on Radiological Protection. Ann ICRP (2003) 33:1–117. 10.1016/S0146-6453(03)00024-1 14614921

[27] Chaudhary P Marshall TI Currell FJ Kacperek A Schettino G Prise KM. Variations in the Processing of DNA Double-Strand Breaks Along 60-Mev Therapeutic Proton Beams. Int J Radiat Oncol Biol Phys (2016) 95:86–94. 10.1016/j.ijrobp.2015.07.2279 26452569 PMC4840231

[28] Goodhead DT Bance DA Stretch A Wilkinson RE. A Versatile plutonium-238 Irradiator for Radiobiological Studies With Alpha-Particles. Int J Radiat Biol (1991) 59:195–210. 10.1080/09553009114550181 1671067

[29] Tracy BL Stevens DL Goodhead DT Hill MA. Variation in RBE for Survival of V79-4 Cells as a Function of Alpha-Particle (Helium Ion) Energy. Radiat Res (2015) 184:33–45. 10.1667/RR13835.1 26121227

[30] Nickson CM Moori P Carter RJ Rubbi CP Parsons JL. Misregulation of DNA Damage Repair Pathways in HPV-Positive Head and Neck Squamous Cell Carcinoma Contributes to Cellular Radiosensitivity. Oncotarget (2017) 8:29963–75. 10.18632/oncotarget.16265 PMC5444717 28415784

[31] Edmonds MJ Carter RJ Nickson CM Williams SC Parsons JL. Ubiquitylation-Dependent Regulation of NEIL1 by Mule and TRIM26 Is Required for the Cellular DNA Damage Response. Nucleic Acids Res (2017) 45:726–38. 10.1093/nar/gkw959 PMC5314803 27924031

[32] Sutherland BM Bennet PV Sidorkina O Laval J. Clustered DNA Damages Induced in Isolated DNA and in Human Cells by Low Doses of Ionizing Radiation. Proc Natl Acad USA (2000) 97:103–8. 10.1073/pnas.97.1.103 PMC26623 10618378

[33] Sutherland BM Bennett PV Sutherland JC Laval J. Clustered DNA Damages Induced by X Rays in Human Cells. Radiat Res (2002) 157:611–6. 10.1667/0033-7587(2002)157[0611:CDDIBX]2.0.CO;2 12005538

[34] Fabbrizi MR Hughes JR Parsons JL. The Enzyme-Modified Neutral Comet (Emnc) Assay for Complex Dna Damage Detection. Methods Protoc (2021) 4:14. 10.3390/mps4010014 33669320 PMC7931015

[35] Skowyra A Allan LA Saurin AT Clarke PR. Usp9x Limits Mitotic Checkpoint Complex Turnover to Strengthen the Spindle Assembly Checkpoint and Guard Against Chromosomal Instability. Cell Rep (2018) 23:852–65. 10.1016/j.celrep.2018.03.100 PMC5917450 29669289

[36] Peddaboina C Jupiter D Fletcher S Yap JL Rai A Tobin RP. The Downregulation of Mcl-1 Via USP9X Inhibition Sensitizes Solid Tumors to Bcl-xl Inhibition. BMC Cancer (2012) 12:541. 10.1186/1471-2407-12-541 23171055 PMC3543233

[37] Habata S Iwasaki M Sugio A Suzuki M Tamate M Satohisa S. BAG3-Mediated Mcl-1 Stabilization Contributes to Drug Resistance Via Interaction With USP9X in Ovarian Cancer. Int J Oncol (2016) 49:402–10. 10.3892/ijo.2016.3494 27120977

[38] Cui J Sun W Hao X Wei M Su X Zhang Y. EHMT2 Inhibitor BIX-01294 Induces Apoptosis Through PMAIP1-USP9X-MCL1 Axis in Human Bladder Cancer Cells. Cancer Cell Int (2015) 15:4. 10.1186/s12935-014-0149-x 25685062 PMC4326523

[39] Trivigno D Essmann F Huber SM Rudner J. Deubiquitinase USP9x Confers Radioresistance Through Stabilization of Mcl-1. Neoplasia (2012) 14:893–904. 10.1593/neo.12598 23097624 PMC3479835

[40] Wolfsperger F Hogh-Binder SA Schittenhelm J Psaras T Ritter V Bornes L. Deubiquitylating Enzyme USP9x Regulates Radiosensitivity in Glioblastoma Cells by Mcl-1-Dependent and -Independent Mechanisms. Cell Death Dis (2016) 7:e2039. 10.1038/cddis.2015.405 26775694 PMC4816183

[41] Grasso D Ropolo A Lo Re A Boggio V Molejon MI Iovanna JL. Zymophagy, a Novel Selective Autophagy Pathway Mediated by VMP1-USP9x-p62, Prevents Pancreatic Cell Death. J Biol Chem (2011) 286:8308–24. 10.1074/jbc.M110.197301 PMC3048716 21173155

[42] Lu Q Zhang FL Lu DY Shao ZM Li DQ. USP9X Stabilizes BRCA1 and Confers Resistance to DNA-Damaging Agents in Human Cancer Cells. Cancer Med (2019) 8:6730–40. 10.1002/cam4.2528 PMC6825982 31512408

[43] O’dea R Santocanale C. Non-Canonical Regulation of Homologous Recombination DNA Repair by the USP9X Deubiquitylase. J Cell Sci (2020) 133:jcs.233437. 10.1242/jcs.233437 31964704

[44] Hori A Toda T. Regulation of Centriolar Satellite Integrity and its Physiology. Cell Mol Life Sci (2017) 74:213–29. 10.1007/s00018-016-2315-x PMC5219025 27484406

[45] Odabasi E Gul S Kavakli IH Firat-Karalar EN. Centriolar Satellites Are Required for Efficient Ciliogenesis and Ciliary Content Regulation. EMBO Rep (2019) 20:e47723. 10.15252/embr.201947723 31023719 PMC6549029

[46] Villumsen BH Danielsen JR Povlsen L Sylvestersen KB Merdes A Beli P. A New Cellular Stress Response That Triggers Centriolar Satellite Reorganization and Ciliogenesis. EMBO J (2013) 32:3029–40. 10.1038/emboj.2013.223 PMC3844950 24121310

[47] Loffler H Fechter A Liu FY Poppelreuther S Kramer A. DNA Damage-Induced Centrosome Amplification Occurs Via Excessive Formation of Centriolar Satellites. Oncogene (2013) 32:2963–72. 10.1038/onc.2012.310 22824794

[48] Cancer Genome Atlas N. Comprehensive Genomic Characterization of Head and Neck Squamous Cell Carcinomas. Nature (2015) 517:576–82. 10.1038/nature14129 PMC4311405 25631445

